# Idiopathic Inflammatory Superior Mesenteric Artery Aneurysm: Case Report and Systematic Literature Review

**DOI:** 10.1016/j.ejvsvf.2025.08.005

**Published:** 2025-09-04

**Authors:** Candice Vallauri, Salomé Kuntz, Anne Lejay, Nabil Chakfé

**Affiliations:** a Department of Vascular Surgery, Kidney Transplantation and Innovation, University Hospital of Strasbourg, Strasbourg, France; b Gepromed, Strasbourg, France; c Department of Physiology, University Hospital of Strasbourg, France

**Keywords:** aneurysm, superiorir mesenteric artery, inflammatory

## Abstract

**Introduction:**

Superior mesenteric artery aneurysm (SMAA) is a rare presentation mostly secondary to atherosclerosis, infection, cystic medial dysplasia, collagen vascular disorders, trauma, and arterial dissection. However, inflammatory SMAAs are sometimes idiopathic. The aim of the article is to report the case of an idiopathic inflammatory SMAA and perform a systematic literature review.

**Case report:**

A 61 year old man presented with chronic abdominal pain. Computed tomography angiography showed a typical 65 mm diameter inflammatory abdominal aortic aneurysm and a SMAA with similar signs of wall inflammation. Positron emission tomography computed tomography confirmed the inflammation by active fixation. A full workup including all inflammatory markers was negative, and no cause was found. Treatment consisted of fenestrated endograft implantation. At 11 months, follow up confirmed aneurysm exclusion and patency of the stents and a similar SMAA diameter with some mild remaining inflammation.

**Literature review:**

A systematic review of the MEDLINE and PubMed databases extending from 1979 to 2024 by a combined strategy of Medical Subject Headings terms returned only two publications of idiopathic inflammatory SMAA in two patients. In both cases, the symptoms were abdominal or lower back pain and the diagnosis was made by computed tomography angiography. Treatment was open surgery in one case and medical in the other. Median follow up was 18 months.

**Conclusion:**

Idiopathic inflammatory SMAA is rarely described in the literature. The aetiological assessment including histological data should be exhaustive in order not to miss a differential diagnosis.

## Highlights

• Rare idiopathic inflammatory SMA aneurysm successfully managed by FEVAR.
• Systematic review identified only two similar idiopathic inflammatory SMA cases.
• PET-CT helped confirm inflammatory nature and guide treatment strategy.
• Endovascular repair maintained patency with stable imaging at 11 months.

## INTRODUCTION

Superior mesenteric artery aneurysms (SMAAs) represent 5.5% of all visceral aneurysms.^1^ The main aetiologies are atherosclerosis, infection, cystic medial dysplasia, collagen vascular disorders, trauma, inflammation, arterial dissection, and idiopathic.^2^ The main feared complication is life threatening rupture. Inflammatory SMAA is a rare presentation, and the standard of care for its diagnosis and treatment has not yet been established.^2^ The aim of this article is to report a case of an idiopathic inflammatory SMAA and perform a systematic literature review of this pathology.

## CASE REPORT

**Figure 1:**
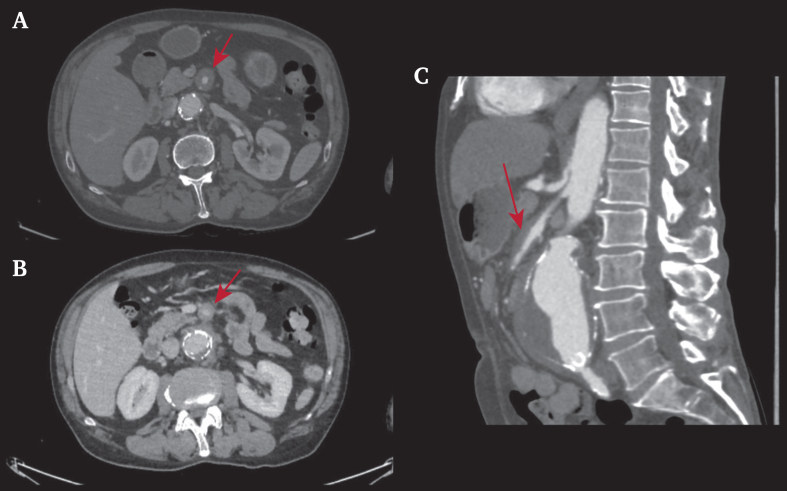
Computed tomography angiography The red arrows indicate the inflammatory segment of the superior mesenteric artery.

**Figure 2:**
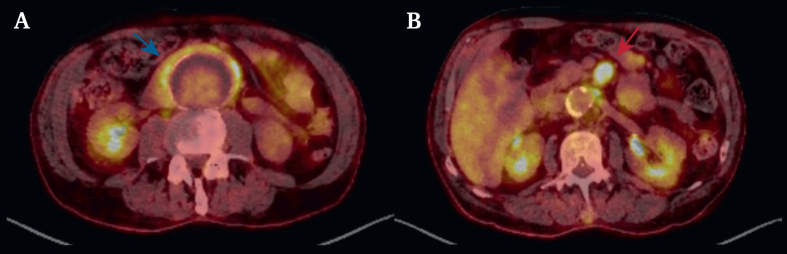
Inflammatory reaction on positron emission tomography computed tomography. The blue arrow highlights the abdominal aortic aneurysm. The red arrow highlights the superior mesenteric artery.

**Figure 3:**
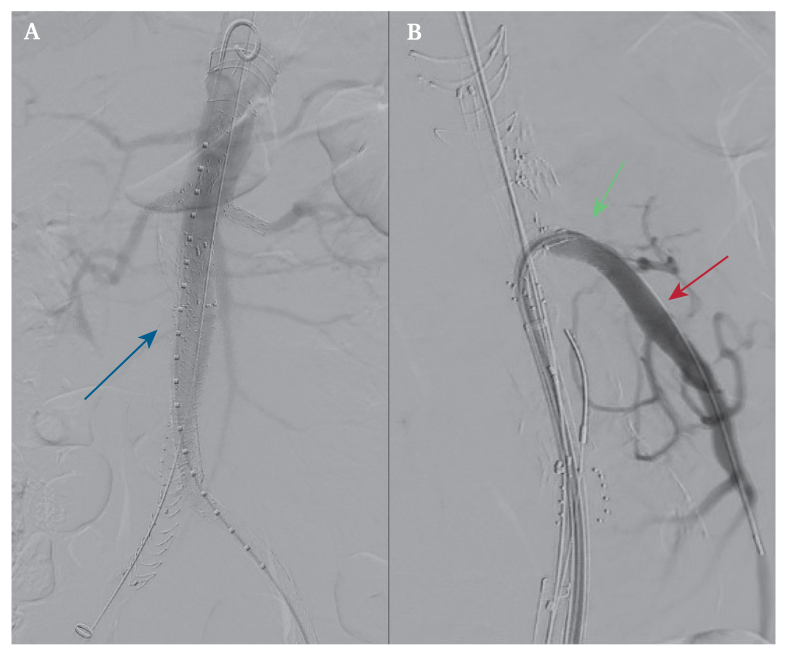
Intra-operative angiography. The blue arrow highlights fenestrated endovascular aortic repair. The red arrow highlights the superior mesenteric artery aneurysm. The green arrow highlights the superior mesenteric artery stent.

**Figure 4:**
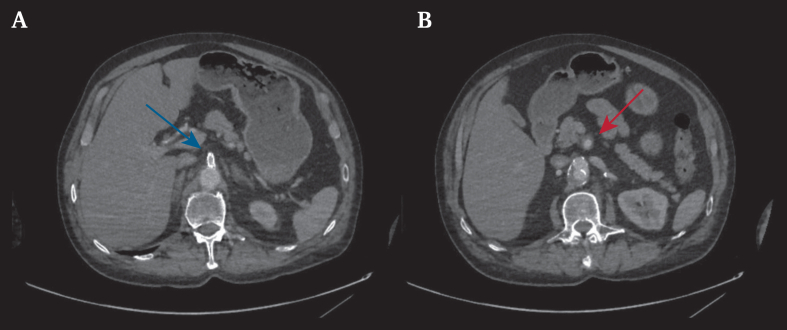
Computed tomography angiography 11 months after surgery. The blue arrow highlights the superior mesenteric artery stent. The red arrow highlights the superior mesenteric artery.

A 61 year old man presented with an abdominal aortic aneurysm (AAA) diagnosed on duplex ultrasound performed for abdominal pain. He had a history of arterial hypertension, dyslipidaemia, active smoking, and left common iliac artery angioplasty and stenting. Computed tomography angiography (CTA) revealed a 65 mm diameter inflammatory AAA associated with a 16 mm SMAA (Fig. 1). It also revealed vessel wall thickening and peri-vessel inflammation. Blood tests revealed a normal white blood cell count of 9.24 g/L and slight increase in C reactive protein of 6.9 mg/L. Positron emission tomography computed tomography (PET-CT) revealed hypermetabolism in the AAA and SMAA walls (Fig. 2). An extensive aetiological workup carried out in association with the departments of vascular medicine, internal medicine, and rheumatology excluded the diagnosis of infection, collagen vascular disorder, and immune disease within the limits of the absence of histological analysis. The tests carried out to eliminate an infective origin or a systemic disease included blood cultures, serologies (syphilis, hepatitis B virus, hepatitis C virus, human immunodeficiency virus, *Bartonella*, *Coxiella*), protein electrophoresis, and a search for antineutrophil cytoplasmic auto-antibody associated vasculitis and immunoglobulin G4 disease. After a multidisciplinary discussion, both surgical options were considered, and endovascular aortic treatment was chosen in collaboration with the patient. The SMAA was not treated by itself because the size of the aneurysm did not meet the treatment criteria,^3^ and monitoring of the lesion was chosen without previous corticoid treatment. In the absence of a proximal neck, implantation of a Fenestrated Anaconda endograft (Terumo Aortic, Inchinnan, Scotland, United Kingdom) was performed with four fenestrations (coeliac trunk, superior mesenteric, and both renal arteries; Fig. 3). The patient was discharged the day after the operation. Follow up CTA at 11 months revealed no endoleak with all stents patent and a similar SMAA diameter with some mild inflammation (Fig. 4).

## LITERATURE REVIEW

### Methods

The MEDLINE and PubMed databases were searched from 1979 to 2024 using a search strategy of Medical Subject Headings terms. The Medical Subject Headings terms used were ‘arteries, superior mesenteric’, ‘aneurysm’, and ‘inflammatory’. All titles and abstracts identified with the search strategy were screened for relevance by two reviewers (CV and SK) to include only those about idiopathic SMAA. The same reviewers independently extracted data using a standardised form. This was done in duplicate to increase accuracy and to reduce measurement bias. Any disagreement in data collection was resolved by consensus. Full texts of all relevant articles were obtained and reviewed for suitability. The reference lists of each article were scanned for other potentially relevant studies. Studies included English and French full texts. All studies reporting on mycotic aneurysms, collagen vascular disorders, and infected aneurysms were excluded.

### Results

**Figure 5:**
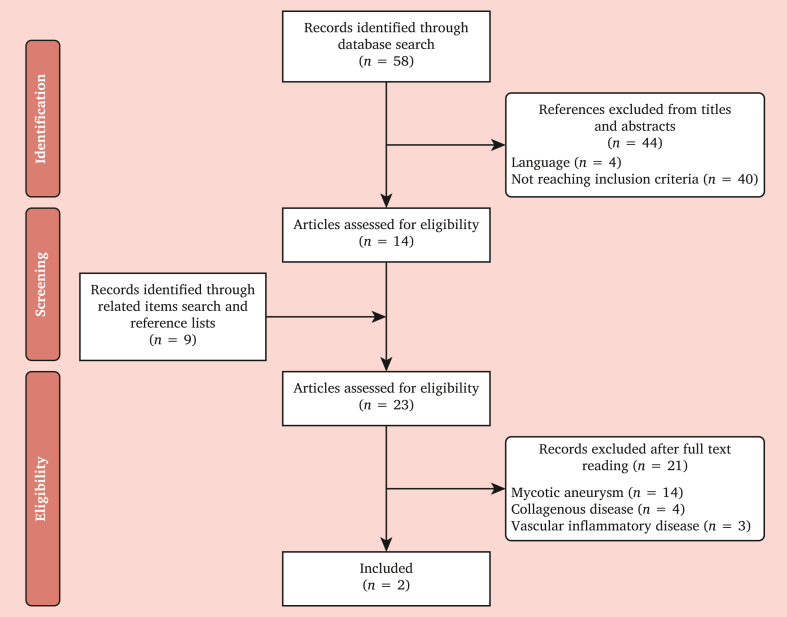
Flowchart showing study selection.

The systematic search identified two single case reports^4^^,^^5^ (Fig. 5 and Table 1). Two patients were included. The ages of the patients were 28 and 64 years. Both patients were men. Symptoms included abdominal pain with no fever in both cases. Both had CTAs. The diameters of the aneurysms were 36 mm and 30 mm. Angiography was performed in one case. No PET-CT was performed.

**Table 1:** Systematic review: case reports.

Author	Year	Sex	Age – y	Diagnosis	Imagery	Diameter – cm	Biology	Infection	Antibiotic therapy	Genetics or syndrome	Immunotherapy	Surgery	Histology	Follow up – y	Complications
Jiang *et al.*^5^	2022	M	28	Inflammatory SMA aneurysm	CT scan + angiography	3.6	CRP elevation				Methylprednisolone + prednisone			1	
Armstrong and Franklin^4^	2006	M	64	Inflammatory SMA branch aneurysm + no coeliac trunk	CT scan	3						Aneurysmectomy and pancreaticoduodenal artery reconstruction with an interposition vein graft from the SMA		2	

CRP = C reactive protein; CT = computed tomography; M = male; SMA = superior mesenteric artery.

One case was treated by aneurysmectomy with vein graft reconstruction, and the other with immunotherapy. Intravenous corticosteroids were used for three days, which were then replaced by oral administration and continued long term. Pain disappeared after two days of medical treatment, and the SMAA thrombosed with an ileocolic artery supplied by collateral circulation. Follow up was one and two years, with no complications.

## DISCUSSION

This literature review shows that little is known about idiopathic inflammatory SMAA. Only two cases have been reported, each with a different treatment. SMAA has multiple aetiologies, which determine the treatment.

Inflammatory SMAA could be linked to genetic disorders or vascular inflammatory diseases. Infection must be ruled out as well. The tests carried out to eliminate an infective origin or a systemic disease should include the search for an inflammatory syndrome, blood cultures, serologies, protein electrophoresis, and antineutrophil cytoplasmic auto-antibody associated vasculitis and immunoglobulin G4 disease. Through the literature review, other inflammatory SMAAs were found.^6^, ^7^, ^8^, ^9^, ^10^, ^11^, ^12^, ^13^ Clinical presentations were broad and included abdominal or lower back pain and fever.^6^, ^7^, ^8^^,^^10^, ^11^, ^12^, ^13^ Discovery might be incidental but can also be life threatening with rupture. Aetiologies included genetics^7^, ^8^, ^9^^,^^12^ (mostly Ehlers–Danlos syndrome), infective with cholecystitis^13^ or spondylodiscitis,^11^ and inflammatory such as Behçet disease,^10^ Takayasu disease,^11^ and rheumatoid arthritis. Definitive aetiology is hard to prove given the lack of histological analysis.

Imaging modalities included mostly CTA, and surprisingly, no PET-CT was performed. Treatment often included surgery: aneurysmectomy and vein reconstruction,^8^^,^^13^ aneurysmorrhaphy,^9^ and three endovascular procedures (two embolisations^10^^,^^12^ and stenting^7^).

PET-CT was used to confirm the inflammatory nature of the SMAA and rule out other active sites. Notably, the inflammatory SMAA did not alter AAA endovascular treatment but was a good argument for choosing an endovascular option. Superior mesenteric artery stenting had no effect on inflammation.

Optimal management is still debated. The guidelines for visceral aneurysms recommend surgical management from a diameter of 25 mm but do not consider the aetiology.^4^ However, for certain aetiologies, such as systemic inflammatory diseases, additional medical therapy seems necessary and often comes down to corticosteroid therapy. For surgical management, open or endovascular surgery appears similar in follow up and complications. However, regarding genetic causes, such as collagenosis, open surgical management may be preferable in view of aortic guidelines^14^ and the case of post-stenting rupture found in the literature.^7^ Managing inflammatory SMAA requires addressing its cause through multidisciplinary care.

## CONCLUSION

Idiopathic inflammatory SMAA is hardly described in the literature. More data are needed to offer the best management for this disease, especially histological findings. Aetiological assessment is essential to avoid overlooking a differential diagnosis that would modify the treatment.

## Funding

None.

## CONFLICTS OF INTEREST

None.

## References

[1] Messina L.M., Shanley C.J. (1997). Visceral artery aneurysms. Surg Clin North Am.

[2] Stone W.M., Abbas M., Cherry K.J., Fowl R.J., Gloviczki P. (2002). Superior mesenteric artery aneurysms: is presence an indication for intervention?. J Vasc Surg.

[3] Björck M., Koelemay M., Acosta S., Bastos Goncalves F., Kölbel T., Kolkman J.J. (2017). Editor's choice – management of the diseases of mesenteric arteries and veins: clinical practice guidelines of the European Society of Vascular Surgery (ESVS). Eur J Vasc Endovasc Surg.

[4] Armstrong P.J., Franklin D.P. (2006). Superior mesenteric artery branch aneurysm with absence of the celiac trunk. Vascular.

[5] Jiang J., Liu Y., Ding X. (2022). Successful conservative treatment of an isolated inflammatory superior mesenteric artery aneurysm. Interact Cardiovasc Thorac Surg.

[6] Choo C.H., Yen H.H. (2013). Unusual upper gastrointestinal bleeding: ruptured superior mesenteric artery aneurysm in rheumatoid arthritis. World J Gastroenterol.

[7] de Leeuw K., Goorhuis J.F., Tielliu I.F.J., Symoens S., Malfait F., de Paepe A. (2012). Superior mesenteric artery aneurysm in a 9-year-old boy with classical Ehlers–Danlos syndrome. Am J Med Genet A.

[8] Dorigo W., Pulli R., Innocenti A.A., Anichini C., Azas L., Barbanti E. (2004). Isolated inflammatory aneurysm of superior mesenteric artery: unexpected pathologic diagnosis. J Vasc Surg.

[9] Gomes V.C., Parodi F.E., Wood J.C., Motta F., Farber M.A. (2024). Rare case of abdominal aortic and multiple visceral aneurysms in a pediatric patient with PIK3CA mutation and vasculitis. Vasc Endovascular Surg.

[10] Güven K., Rozanes I., Kayabali M., Minareci O. (2009). Endovascular treatment of a superior mesenteric artery aneurysm secondary to Behcet's disease with Onyx (ethylene vinyl alcohol copolymer). Cardiovasc Intervent Radiol.

[11] Matsumoto T., Ishizuka M., Iso Y., Kita J., Kubota K. (2015). Mini-laparotomy for superior mesenteric artery aneurysm due to Takayasu's arteritis. Int Surg.

[12] Oechsle S., Vollert K., Buecklein W., Michl W., Roemer F.W. (2006). Percutaneous treatment of a ruptured superior mesenteric artery aneurysm in a child. Pediatr Radiol.

[13] Suehiro Y., Seo H., Kubota Y., Suehiro S., Hirai H. (2020). Peripheral inflammatory superior mesenteric artery aneurysm diagnosed by intraoperative and histological findings: a case report. Ann Vasc Dis.

[14] Riambau V., Böckler D., Brunkwall J., Cao P., Chiesa R., Coppi G. (2017). Editor's choice – management of descending thoracic aorta diseases: clinical practice guidelines of the European Society for Vascular Surgery (ESVS). Eur J Vasc Endovasc Surg.

